# Evaluation of the detection of 14 high-risk human papillomaviruses with HPV 16 and HPV 18 genotyping for cervical cancer screening

**DOI:** 10.3892/etm.2013.1309

**Published:** 2013-09-18

**Authors:** MEI-LU BIAN, JIAO-YING CHENG, LI MA, XIAO CONG, JUN LIU, YING CHEN, XI CHEN

**Affiliations:** Department of Gynecology and Obstetrics, China-Japan Friendship Hospital, Beijing 100029, P.R. China

**Keywords:** cervical cancer screening, genotyping, cervical intraepithelial lesions, human papillomavirus

## Abstract

The American Society for Colposcopy and Cervical Pathology (ASCCP) suggests that women ≥30 years old, with a negative cytopathological test but a positive high-risk (HR) human papillomavirus (HPV) test should undergo HPV 16 and HPV 18 genotyping. If this test is positive, immediate cervical pathology is required. Therefore, the aim of this study was to evaluate the effectiveness and clinical value of testing for 14 HR HPVs with HPV 16 and HPV 18 genotyping for cervical cancer (CC) screening. A total of 424 females from the China-Japan Friendship Hospital were selected and randomly divided into two groups (A and B). All participants underwent two different testing methods: the liquid-based cytology test (LCT) and a HPV DNA test. For the HPV DNA test, participants in group A underwent the hybrid capture II (HC-II) testing method while participants in group B were tested using the quantitative polymerase chain reaction (qPCR; HBRT-H14) method. The sensitivity, specificity, positive predictive value and negative predictive value for the detection of cervical intraepithelial neoplasia (CIN) grade II or greater using HBRT-H14 were 96.30, 78.17, 23.21 and 99.68%, respectively. In Group B, compared with other HR HPV types, HPV 16 and HPV 18 infection led to the increased possibility of cervical lesions graded CIN II or higher (8.11 and 51.28%, respectively). A significant difference in the rates of CC and CIN II or higher was observed among women who were i) infected with HPV 16 and/or HPV 18, ii) infected with other HR HPV types and iii) diagnosed as negative for HR HPV infection (χ^2^=93.976, P=0.0001). In conclusion, HBRT-H14 is applicable for CC screening with the advantage of genotyping for HPV 16 and HPV 18, which may help to improve triage management for women with negative cytology.

## Introduction

It has been identified that ~95–100% of the incidences of cervical cancer (CC) are due to infection with the human papillomavirus (HPV) (1–3). There are 15 types of high-risk (HR) HPV that may lead to the development of cervical intraepithelial neoplasia (CIN) and CC (4,5). Over the past 20 years, various studies have stated that the detection of HPV may be used as an auxiliary tool for primary screening in CC prevention (6–10). Furthermore, the International Agency for Research on Cancer (IARC) has proposed the use of HPV detection as a screening option (11). Though 15 types of HR HPV have been linked to CC, studies have shown that >50% of CC cases are caused by HPV 16, and 10–15% are caused by HPV 18 (12,13). Thus HPV 16 and HPV 18 may be detected in ~70% of CC cases (14). In addition, HVP 18 causes >35% of cervical adenocarcinomas, which are difficult to detect using cytological tests (12). Therefore the manual of the American Society for Colposcopy and Cervical Pathology (ASCCP; 2006, 2009 and 2012) suggests that women ≥30 years old, with a negative cytopathological test but a positive HR HPV test should undergo HPV 16 and HPV 18 genotyping. If this test is positive, immediate cervical pathology is required. Patients who are HPV 16/18 negative, but who are HR HPV positive should undergo cytopathology and HR HPV testing again after a further 12 months (15,16). In this study, we evaluated the effectiveness of a real-time PCR method from Hybribio to detect 14 high-risk HPV with HPV 16 and HPV 18 genotyping kit (qPCR; HBRT-H14) for CC screening.

## Materials and methods

### Study population

Between August 15, 2011 and November 10, 2011, a total of 424 females, with a mean age of 39.02±12.00 years (range, 20–55 years), underwent CC screening and treatment in the China-Japan Friendship Hospital cervical cancer treatment center (Beijing, China). None of the recruited patients had exhibited acute inflammation of the reproductive tract. Written informed consent was obtained from all patients. In group A, hybrid capture II (HC-II) and liquid-based cytology testing (LCT) were preformed; in group B, 14 patients underwent HR HPV with HPV 16 and HPV 18 genotyping using the quantitative polymerase chain reaction (qPCR; HBRT-H14) test and LCT. For subjects who were HR HPV positive and had an LCT result that was at least as severe as atypical squamous cells-undetermined significance (ASC-US); HPV 16 and/or HPV 18 positive with negative cytological results; or had a cytological test result that was at least as severe as ASC-US and were HPV negative, colposcopies were performed.

### Cytology test

The thin-layer cytology specimens were obtained using SurePath™ (TriPath Imaging, Becton Dickinson, Burlington, NC, USA), and an experienced gynecologist evaluated the histopathological slides. The Bethesda system (TBS) protocol, revised by the International Cancer Association in 2001, was used as the standard for cytological diagnosis (17). Squamous epithelial abnormalities include ASC [including ASC-US and ASC but excluding high-grade squamous intraepithelial lesions (ASC-H)], low-grade squamous intraepithelial lesion (LSIL), high-grade squamous intraepithelial lesion (HSIL) and squamous cell carcinoma (SCC).

### Detection of 14 HR HPVs with HPV 16 and HPV 18 genotyping and qPCR (HBRT-H14)

HBRT-H14 qPCR is a new PCR assay that detects 12 HR HPV genotypes (HPV 31, 33, 35, 39, 45, 51, 52, 56, 58, 59, 66 and 68) with simultaneous differentiation between HPV 16 and HPV 18. This method was based on the multiplex qPCR assay which utilizes 14 sets of specific primers and four probe sets, designed in the E region of the HPV genome, with varying fluorophores in order to obtain fluorescent detection signals. The channels of reporter dyes FAM, HEX, ROX and Cy5 represent 12 HR HPV genotypes, HPV 16, HPV 18 and the β-globin gene (internal control), respectively. Amplification was performed according to the manufacturer’s instructions (Hybribio Biotechnology Ltd. Corp., Chaozhou, China).

### HC-II for HPV detection

The HC-II test system (Qiagen, Gaithersburg, MD, USA) was used to detect HPV in the obtained samples (HPV 16, 18, 31, 33, 35, 39, 45, 51, 52, 56, 58, 59 and 68) as previously described (18). The 96-well HC-II is able to detect up to 90 clinical samples. Samples require ≥5,000 copies/ml of HR HPV in order to be detected.

### Pathological diagnosis

A cohort that were: HBRT-H14 positive; diagnosed with ASC-US or above; or HPV 16 and/or HPV 18 positive in the HBRT-H14 assay but cytologically negative (n=122) underwent colposcopy testing and cervical biopsies. Multi-point biopsies were performed on the area of epithelial cells to which acetic acid had been applied and on the unstained iodine test point. For the normal transformation zone, four points (2,4,8,10) in total were bioposies. Sherman *et al* (19) reported that the cumulative incidence of CC was 0.79% with negative cytological and HPV test results, and all samples were not deemed to be CIN or CC according to the histological tests. It was assumed that 300 subjects with negative results in the cytological and HPV tests were normal without CIN or CC.

### Statistical analysis

Histopathological results were considered to be the gold standard. SPSS 16.0 (SPSS, Inc., Chicago, IL, USA) was used to calculate the sensitivity, specificity, positive predictive value and negative predictive value of HBRT-H14 for CC screening. Comparisons among groups were made using the χ^2^ test. P<0.05 was considered to indicate a statistically significant difference.

## Results

### Correlation between HBRT-H14 HPV and histopathological test results

Using the HBRT-H14 HPV test and the LCT screening method, 19 samples in 27 subjects with CIN II or above were HPV 16 positive according to the HPV DNA test, accounting for 70.37% (19/27), among which four samples were cytologically negative. One sample (3.70%, 1/27) was identified to be HPV 18 positive with other HR HPV infection; six subjects tested positive for other HR HPV (non-HPV 16 and non-HPV 18), accounting for 22.22% (6/27); and one sample (3.70%, 1/27) gave a negative HR HPV result (Table I). CIN II and above was considered to be pathologically positive with CIN I and below considered to be pathologically negative. For the screening of CIN and CC, the sensitivity of the HBRT-H14 HPV test was 96.30%, the specificity was 78.17%, the positive predictive value was 23.21% and the negative predictive value was 99.68%. Nineteen samples in 34 subjects with HPV 16 positive were CIN II or above (histopathology). However, four samples were HPV positive and cytopathologically negative. The subjects who were graded as CIN II or above comprised one (20%, 1/5) who was HPV 18 positive, six (8.11%, 6/74) who were other HR (non-HPV16 and non-HPV 18) positive and one (0.32%, 1/309) who was HR HPV negative. The risk of cervical precancer and cancer demonstrated a significant difference between HPV 16 positive, HPV 18 positive, other HR HPV (non-HPV 16 and non-HPV 18) positive and HR HPV negative subjects (χ^2^=93.976, P=0.0001).

### Evaluation of the clinical value of the HBRT-H14 HPV test

In order to examine whether the HBRT-H14 HPV test is only required in the CC screening of females ≥30 years old, the subjects were divided into two groups with 30 years of age as the borderline. Table II shows that the HR HPV infection rate was 21.76% (26/120) in group 1 (females <30 years old), and 28.57% (86/301) in group 2 (females ≥30 years old). There was no statistical significance between the two groups (χ^2^=2.09, P>0.05). The HPV 16 infection rates [6.67%, (8/120) and 8.64% (26/301)] also showed no statistically significant difference between the two groups (χ^2^=2.09, P>0.05). In HPV 16 positive subjects, the percentage of patients graded as CIN II or above was 25% (2/8) for group 1 and 65.38% (17/26) for group 2; the difference was deemed to be statistically significant (χ^2^=4.123, P<0.05). Due to the low detection rate of HPV 18, no statistical analysis was performed for this virus. The infection rates of other HR HPV (non-HPV 16 and non-HPV 18) infections were 15.00% (18/120) and 18.60% (56/301) in groups 1 and 2, respectively (χ^2^=0.77, P>0.05); there was no statistically significant difference between the two groups. The rates of other HR HPV (non-HPV 16 and non-HPV 18) infection with CIN II or above are 11.11% (2/18) and 7.14% (4/56) in groups 1 and 2, respectively (χ^2^=0.0016, P>0.05); the difference between groups was considered to have no statistical significance.

### Analysis of cervical cytological, HBRT-H14 and histopathological test results

Of the 379 subjects considered to be normal or inflamed by the cervical cytological method, 18, 5, and 56 subjects, respectively, were tested to be HPV 16, HPV 18 and other HR HPV (non-HPV 16 and non-HPV 18) positive. Histological results following the cervical biopsy were that samples from four of the HPV 16 positive patients, one of the HPV 18 positive patients and none of the patients positive for other HR HPV were graded as CIN II or above. In the nine subjects diagnosed with ASC-US using the cervical cytological method, two were HPV 16 positive and were graded CIN II according to the cervical biopsy; the other seven subjects were both HPV 16 and HPV 18 negative, and evaluated as having inflammation according to the biopsy. Using the cervical cytological test, 17 subjects were diagnosed with LSIL, of which two were HPV 16 positive and graded CIN II by biopsy, and 11 subjects were other HR HPV (non-HPV 16 and non-HPV 18) positive, among which four were graded CIN II. The remaining four subjects were HR HPV negative and one was confirmed to have inflammation, three were confirmed to have CIN I according to the biopsy results (Table III). A grading of CIN II or above was made in 19 of the 34 HPV 16 positive patients, one of the 5 HPV 18 positive patients, six of the 74 other HRHPV (non-HPV 16 and non-HPV 18) positive patients and one of the 309 HR HPV negative subjects. The differences between groups were considered to be statistically significant (χ^2^=93.976, P=0.0001). In cytologically negative subjects diagnosed with ASC-US and LSIL, the differences between the various types of HPV associated with the CC were deemed to be statistically significant (χ^2^=32.742, 9.535 and 7.654, P=0.0001, 0.002 and 0.022, respectively).

### Analysis of HC-II HPV and the cytological method of testing

Table IV shows the correlation between the HC-II HPV and histological results. CIN II and above was considered to be pathologically positive and CIN I and below was considered to be pathologically negative. The sensitivity, specificity, positive predictive value and negative predictive value for the detection of CIN II and above using HC-II HPV and cytological testing were 77.78, 79.35, 20.39 and 98.13%, respectively. In terms of clinical value for the detection of CIN and CC, there was no significant difference between HBRT-H14 and HC-II when used with cytological examination (P<0.05). Furthermore, the sensitivity and the negative predictive value were improved using HBRT-H14.

## Discussion

Analysis of 14 HR HPVs with the 16/18 genotyping real-time PCR kit is based on a multiplex qPCR assay utilizing specific sets of amplification primers with varying fluorophores in order to obtain fluorescent detection signals. qPCR is capable of dispensing with the problems associated with electrophoresis and the probability of contamination from traditional PCR (20). PCR amplification and detection are conducted in the same reaction tube; multiple reaction tubes use four different reporter dyes capable of tracking different targets. The reaction mixture for each sample includes a cellular β-globin gene which is capable of fully monitoring the entire testing process from DNA extraction to signal detection.

In this study, three β-globin negative samples were reported among 424 samples (0.7%), rendering these three subjects invalid. It was possible that the HPV copy number was too low to be amplified due to too few cells being obtained. False negative results in HPV DNA detection are likely to increase if this quality monitoring option is lacking. Thus it is critical to include quality monitoring in HPV detection.

In the natural history of HPV infection, the majority of infections are temporary, particularly among young women. Only a small proportion are persistently infected by HR HPV, and usually CC develops following 10 years of continuous infection (21). Thus, the Food and Drug Administration (FDA) approved that the testing of women ≥30 years old may include HPV DNA detection as a supplementary method of diagnosis (22–24). In the present study, no statistically significant differences were observed in the incidence of HPV 16 infection and of other HR HPV (excluding HPV 16 and 18) infection between the age groups (P>0.05). Statistical analysis was not performed for HPV 18 due to its low infection rate. However, there was a statistically significant difference between the two age groups of HPV 16 positive females (<30 and ≥30 years old), graded CIN II and above (P<0.05). Thus for women ≥30 years old, HPV 16 infection has a very high risk.

Song *et al* (25) considered the HPV DNA test to be a valuable resource for CC screening, triage and the management of patients with ASC-US and LSIL, and post-surgical follow-up for CIN and CC. The clinical dilemma is how to manage female patients who are HR HPV positive but cytologically negative. In the present study, statistically significant differences were identified among the HPV 16 positive, HPV 18 positive, other HR HPV (excluding HPV 16 and 18) positive and HR HPV negative females with regard to a grading of CIN II and above. Thus, the risk factors for CC may be listed as: HPV 16 positive > HPV 18 positive > other HR HPV type positive > HR HPV negative. Our study has shown that the patients may also divided according to whether the type of HPV infection is HPV 16 or HPV 18. Colposcopies should be performed if the females are demonstrated to be HVP 16 and/or HPV 18 positive, whereas subjects who are HPV 16 and HPV 18 negative should be tested again one year later (10,11).

The guidelines from the ASCCP and those of the Spanish CC screening and prevention programs suggest that cytologically negative but HR HPV positive subjects should continue to undergo HPV 16 and HPV 18 genotyping (26). If the HPV 16 or HPV 18 tests are positive, examination by colposcopy is required. Therefore, the HBRT-H14 screening method that detects 14 HR HPV types, including the specific detection of HPV 16/18 genotypes, is likely to save time. Furthermore, HPV 18 infection related to certain cervical lesions is difficult to detect using cytological methods (for example cervical adenocarcinoma). In our study, five cases were HPV 18 positive. These cases included one that was diagnosed as inflammation by cervical cytological testing but was demonstrated to be cervical adenocarcinoma by biopsy. Therefore, it is not sufficient to report the infection of HR HPVs without classification; HPV 16 and HPV 18 genotyping should also be performed. Our study indicated with other HR-HPV, HPV 16 and HPV 18 had a higher risk of causing CC. The results of the current study were similar to those of Wong *et al* (27).

## Figures and Tables

**Table I.** HBRT-H14 detection result and its association with histopathological studies.

HPV results	Total (n)	Histopathologically negative (n)	CIN I (n)	CIN II (n)	CIN III (n)	CA (n)
HPV 16 (+)	34^a^	13^a^	2	11	5	3
HPV 18 (+)	5^a^	4^a^	0	0	0	1
Other (+)^b^	74	65	3	5	0	1
HPV DNA (−)	309	305	3	0	1	0
Total	421	386	8	16	6	5

a One subject was both HPV 16 and HPV 18 positive, and was demonstrated to have inflammation according to the pathological test.

b The HPV 16 positive group includes subjects with HPV 16 and other HPV types, and the HPV 18 positive group includes subjects with HPV 18 and other HPV types; therefore, this group is non-HPV 16, non-HPV 18 and other HR HPV positive. HPV, human papillomavirus; CIN, cervical intraepithelial neoplasia; CA, carcinoma; HR, high risk.

**Table II.** HPV infection in women <30 and ≥30 years old.

CIN grade	<30 years old					≥30 years old				
	HPV 16 (+)	HPV 18 (+)	Other (+)^b^	Negative	Total	HPV 16 (+)	HPV 18 (+)	Other (+)^b^	Negative	Total
≤CIN I	6	0	16	94	116	9^a^	4^a^	52	214	278
≥CIN II	2	0	2	0	4	17	1	4	1	23
Total	8	0	18	94	120	26^a^	5^a^	56	215	301

a One subject was both HPV 16 and HPV 18 positive, and was demonstrated to have inflammation according to the pathological test.

b The HPV 16 positive group includes subjects with HPV16 and other HPV types, and the HPV 18 positive group includes subjects with HPV 18 and other HPV types; therefore, this group is non-HPV 16, non-HPV 18 and other HR HPV positive. HPV, human papillomavirus; CIN, cervical intraepithelial neoplasia; HR, high risk.

**Table III.** Correlation between HR HPV, cervical cytological and histological results.

HPV	LCT	Pathological results					
		Normal or inflammation	CIN I	CIN II	CIN III	CA	n
HPV 16 (+)^a^	Normal or inflammation	13^a^	1	4	0	0	18
	ASC-US	0	0	2	0	0	2
	LSIL	0	0	0	2	0	2
	HSIL	0	1	4	3	3	11
	ASC-H	0	0	1	0	0	1
	n	13	2	11	5	3	34
HPV 18 (+)^a^	Normal or inflammation	4^a^	0	0	0	1	5
Other (+)^b^	Normal or inflammation	56	0	0	0	0	56
	ASC-US	4	0	0	0	0	4
	LSIL	4	3	4	0	0	11
	HSIL	1	0	1	0	1	3
	n	65	3	5	0	1	74
HR HPV (−)	Normal or inflammation	300	0	0	0	0	300
	ASC-US	3	0	0	0	0	3
	LSIL	1	3	0	0	0	4
	HSIL	0	0	0	1	0	1
	AGC	1	0	0	0	0	1
	n	305	3	0	1	0	309
Total	N	386	8	16	6	5	421

a One subject was both HPV 16 and HPV 18 positive, and was demonstrated to have inflammation according to histological testing.

b The HPV 16 positive group includes subjects with HPV 16 and other HPV types, and the HPV 18 positive group includes subjects with HPV 18 and other HPV types; therefore, this group is non-HPV 16, non-HPV 18 other HR HPV positive. HPV, human papillomavirus; LCT, liquid-based cytological technology; CIN, cervical intraepithelial neoplasia; CA, carcinoma; ASC-US, atypical squamous cells-undetermined significance; LSIL, low-grade squamous intraepithelial lesions; HSIL, high-grade squamous intraepithelial lesions; ASC-H, atypical squamous cells-cannot exclude HSIL; AGC, atypical glandular cell; HR, high risk.

**Table IV.** Correlation between HC-II HPV test results and cervical histological results.

HPV	Negative	Pathological results				
		CIN I	CIN II	CIN III	CA	n
HC-II (+)	77	5	11	5	5	103
HC-II (−)	312	3	5	1	0	321
Total	389	8	16	6	5	424

HPV, human papillomavirus; CIN, cervical intraepithelial neoplasia; CA, carcinoma; HC-II, hybrid capture-II.

## References

[1] de Villiers EM, Fauquet C, Broker TR, Bernard HU, zur Hausen H (2004). Classification of papillomaviruses. Virology.

[2] Mahmud SM, Franco EL (2004). An overview of epidemiological and public health research on HPVs presented at the 21st International Papillomavirus Conference in Mexico City, 20–26 February 2004. Papillomavirus Rep.

[3] Kitchener HC, Almonte M, Thomson C (2009). HPV testing in combination with liquid-based cytology in primary cervical screening (ARTISTIC): a randomised controlled trial. Lancet Oncol.

[4] McLachlin CM (2000). Human papillomavirus in cervical neoplasia. Role, risk factors, and implications. Clin Lab Med.

[5] Smith JS, Lindsay L, Hoots B, Keys J, Franceschi S, Winer R, Clifford GM (2007). Human papillomavirus type distribution in invasive cervical cancer and high-grade cervical lesions: a meta-analysis update. Int J Cancer.

[6] Bulkmans NW, Berkhof J, Rozendaal L (2007). Human papillomavirus DNA testing for the detection of cervical intraepithelial neoplasia grade 3 and cancer: 5-year follow-up of a randomised controlled implementation trial. Lancet.

[7] Dillner J, Rebolj M, Birembaut P (2008). Long term predictive values of cytology and human papillomavirus testing in cervical cancer screening: joint European cohort study. BMJ.

[8] Mayrand MH, Duarte-Franco E, Rodrigues I, Canadian Cervical Cancer Screening Trial Study Group (2007). Human papillomavirus DNA versus Papanicolaou screening tests for cervical cancer. N Engl J Med.

[9] Naucler P, Ryd W, Törnberg S (2007). Human papillomavirus and Papanicolaou tests to screen for cervical cancer. N Engl J Med.

[10] Castle PE, Sadorra M, Lau T, Aldrich C, Garcia FA, Kornegay J (2009). Evaluation of a prototype real-time PCR assay for carcinogenic human papillomavirus (HPV) detection and simultaneous HPV genotype 16 (HPV16) and HPV18 genotyping. J Clin Microbiol.

[11] Wright TC, Massad LS, Dunton CJ, 2006 American Society for Colposcopy and Cervical Pathology-sponsored Consensus Conference (2007). 2006 consensus guidelines for the management of women with abnormal cervical cancer screening tests. Am J Obstet Gynecol.

[12] Bosch FX, de Sanjosé S (2003). Chapter 1: Human papillomavirus and cervical cancer - burden and assessment of causality. J Natl Cancer Inst Monogr.

[13] Clifford GM, Smith JS, Plummer M, Muñoz N, Franceschi S (2003). Human papillomavirus types in invasive cervical cancer worldwide: a meta-analysis. Br J Cancer.

[14] Muňoz N, Bosch X, de Sanjosé S (2003). Epidemiologic classification of Human Papilloma Virus types associated with cervical cancer. N Engl J Med.

[15] Poljak M, Ostrbenk A, Seme K (2011). Comparison of clinical and analytical performance of the Abbott realtime high risk HPV test to the performance of hybrid capture 2 in population-based cervical cancer screening. J Clin Microbiol.

[16] Schiffman M, Wentzensen N, Wacholder S, Kinney W, Gage JC, Castle PE (2011). Human papillomavirus testing in the prevention of cervical cancer. J Natl Cancer Inst.

[17] Apgar BS, Zoschnick L, Wright TC (2003). The 2001 Bethesda System terminology. Am Fam Physician.

[18] Ermel A, Qadadri B, Morishita A (2010). Human papillomavirus detection and typing in thin prep cervical cytologic specimens comparing the Digene Hybrid Capture II Assay, the Roche Linear Array HPV Genotyping Assay, and the Kurabo GeneSquareMicroarray Assay. J Virol Methods.

[19] Sherman ME, Lorincz AT, Scott DR (2003). Baseline cytology, human papilomavirus testing, and risk for cervical neoplasia: a 10-year cohort analysis. J Natl Cancer Inst.

[20] Gage JC, Sadorra M, Lamere BJ, PaP Cohort Study Group (2012). Comparison of the cobas human papillomavirus (HPV) test with the hybrid capture 2 and linear assay HPV DNA tests. J Clin Microbiol.

[21] Burd EM (2003). Human papillomavirus and cervical cancer. Clin Microbiol Rev.

[22] Kaliterna V, Lepej SZ, Vince A (2009). Comparison between the Abbott RealTime high risk HPV assay and the Hybrid Capture 2 assay for detecting high-risk human papillomavirus DNA in cervical specimens. J Med Microbiol.

[23] Kovacic MB, Castle PE, Herrero R (2006). Relationships of human papillomavirus type, qualitative viral load, and age with cytologic abnormality. Cancer Res.

[24] Lapierre SG, Sauthier P, Mayrand MH (2012). Human papillomavirus (HPV) DNA triage of women with atypical squamous cells of undetermined significance with cobas 4800 HPV and Hybrid Capture 2 test for detection of high-grade lesions of the uterine cervix. J Clin Microbiol.

[25] Song SH, Hong JH, Kwak SH, Lee JK, Kim MK (2012). Clinical performance assessment of five human papillomavirus DNA tests using liquid-based cytology samples. J Obstet Gynaecol Res.

[26] Cortes J, Martinón-Torres F, Ramony Cajal JM (2010). Primary and secondary prevention of cervical cancers and vulva: recommendations for clinical practice. Prog Obstet Gynecol.

[27] Wong AA, Fuller J, Pabbaraju K, Wong S, Zaharadia G (2012). Comparison of the hybrid capture 2 and cobas 4800 tests for detection of high-risk human papillomavirus in specimens collected in PreservCyt medium. J Clin Microbiol.

